# Acute Respiratory Failure Requiring Invasive Ventilation in Adults With Congenital Syringomyelia/Arnold-Chiari Malformations: A Systematic Review

**DOI:** 10.7759/cureus.70109

**Published:** 2024-09-24

**Authors:** Divakar Hamal, André Fernandes, Prajwal Ghimire, Adrian Wong

**Affiliations:** 1 Critical Care Medicine, King’s College Hospital NHS Foundation Trust, London, GBR; 2 Trauma and Orthopedics, Lewisham and Greenwich NHS Trust, London, GBR; 3 Neurological Surgery, King’s College Hospital NHS Foundation Trust, London, GBR

**Keywords:** arnold-chiari malformation, invasive mechanical ventilation, respiratory failure, syrinx, syringomyelia

## Abstract

Arnold-Chiari malformations (ACM) and congenital syringomyelia/syrinx are rare neurological phenomenons that can present as acute respiratory failure and contribute to multiple extubation failures despite surgical intervention. A systematic review was conducted to scrutinize the current literature, screening 65 papers and including 12 papers (13 patients). Sixty-one percent of patients had type 1 ACM and 70% had a congenital syringomyelia. Neurosurgical intervention occurred in seven patients, five patients had at least one extubation failure which was due to apnea or reoccurrence of respiratory failure, and eight patients needed tracheostomies. The neurosurgical intervention aims to improve patient symptoms, but our data and current literature suggest that patients with these pathologies still undergo long ventilation weans and are not liberated from the ventilator due to ongoing respiratory failure.

## Introduction and background

Acute respiratory failure often leads to emergency department visits, but immediate invasive ventilation is rarely needed unless the cause is clear. Undiagnosed neurological pathology should be considered when these patients are admitted to intensive care and experience extubation failures of unknown etiology. This includes conditions such as Arnold-Chiari malformations (ACM) and congenital syringomyelia [1]. ACMs are genetic deformities of the hindbrain (including the cerebellum) that can lead to neurological and respiratory symptoms that present insidiously in adulthood. In contrast, a congenital syringomyelia/syrinx is a fluid-filled cyst within the spinal cord, primarily caused by ACM but also caused by spinal tumors or other neurological pathologies [1]. ACMs can lead to CSF disruption at the level of the cervical-medullary junction which leads to brainstem compression hindering the vital respiratory centers and increasing the risk of respiratory arrest [2]. Furthermore, congenital syringomyelia (including those caused by ACM) increases CSF accumulation within the spinal cord and, particularly if it occurs in the cervical region, can impair diaphragmatic function leading to impaired respiratory drive and subsequent respiratory failure. Respiratory decompensation from both these pathologies is usually a sign of neurological disease progression or failure of previous management (including surgical interventions) [2].

## Review

Methods

A systematic review was conducted by two independent reviewers across four different databases - Cochrane Library, PubMed, MEDLINE, and Embase - in February 2024, as well as through snowballing techniques from reference lists for other relevant papers. Search terms included “syringomyelia” OR “arnold chiari malformation” AND “respiratory failure” (likewise with MeSH terms in Cochrane Library). The inclusion criteria were adult patients (18 years and older), studies in English, non-clinical trials, and studies published from January 2000 to February 2024. We commonly excluded patients who didn’t have a diagnosed Arnold-Chiari malformation or syringomyelia, and those who didn’t have a documented acute respiratory failure requiring invasive ventilation. Article discrepancies were resolved by a third reviewer with mutual consent. Data were subsequently extracted into a template designed in Microsoft Excel 2020 (Redmond, WA: Microsoft Corp.) with data on the following: patient demographics, past medical and surgical history, initial presenting symptoms, Arnold-Chiari malformation type and syringomyelia presence, diagnostic interventions, surgical intervention, critical care morbidity, and mortality factors inputted onto our template.

The systematic review initially identified a total of 59 studies (after duplicates were reviewed) which were analyzed based on their title, abstract, and full text. A total of 10 studies were initially excluded due to inaccessibility, not available in English, and full paper unavailable (abstract only). This left 49 articles for full-text eligibility which were then assessed by two independent reviewers. Thirty-seven articles were further excluded due to the majority being another neurological pathology, relating to chronic respiratory failure with no invasive ventilation recorded, surgical technique alone, radiological (pictorial) reviews, and pediatric cases. This left 12 studies for full inclusion, which consisted mostly of case reports but also included one literature review and one study with two eligible cases, resulting in a total of 13 patients to analyze.

**Figure 1:**
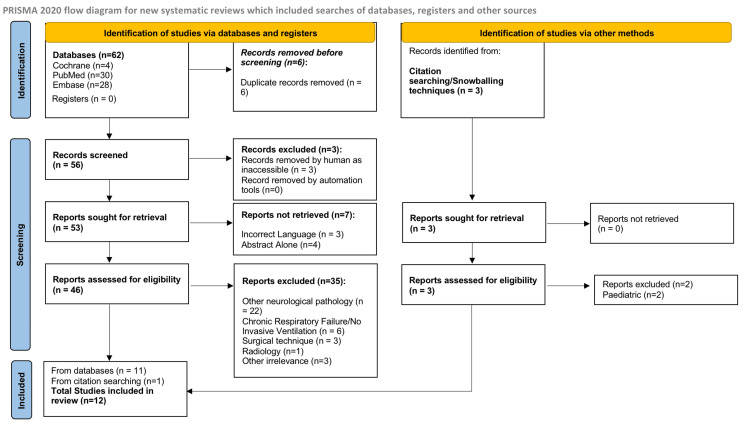
The Preferred Reporting Items for Systematic Reviews and Meta-Analysis (PRISMA) 2020 flow diagram depicting the study selection process. The image is taken from Page et al. [3] and licensed under CCBY 4.0.

Our systematic review was conducted with Preferred Reporting Items for Systematic Reviews and Meta-Analyses (PRISMA) guidelines [3]. The systematic review is registered with the International Prospective Register of Systematic Reviews (PROSPERO) with registration number CRD42024554994. Our screening process is shown in the flow diagram pictorially below (Figure 1).

We also used a risk assessment tool named Risk of Bias in Non-randomized Studies - Of Interventions (ROBINS-I), which is a validated tool used to assess the risk of bias in systematic reviews. A total of two reviewers (DH and AF) conducted the quality assessment of the individual studies and any disputes were discussed and settled by a third senior author (AW). All the signaling questions were answered in full and no potential concerns regarding the overall risk of bias in all the studies arose (Table 1) [4].

**Table 1:** Risk of Bias in Non-randomized Studies - Of Interventions (ROBINS-I) assessment with signaling questions summary.

Study	Bias due to confounding	Bias in selection of participants into the study	Bias in classification of interventions	Bias due to deviations from intended interventions	Bias due to missing data	Bias in measurement of outcomes	Bias in selection of the reported result	Overall bias
Kijsirichareanchai et al. 2014 [1]	Low	Low	Low	Low	Low	Low	Low	Low
Khatib et al. 2020 [2]	Low	Low	Low	Low	Mod	Low	Low	Low
Wani et al. 2011 [5]	Low	Low	Low	Low	Low	Low	Low	Low
Gentry et al. 2001 [6]	Low	Low	Low	Low	Low	Low	Low	Low
Gladding and Whyte 2005 [7]	Low	Low	Low	Low	Low	Low	Low	Low
Fuller and Stanners 2000 [8]	Low	Low	Low	Low	Low	Low	Low	Low
Ding et al. 2020 [9]	Low	Low	Low	Low	Low	Low	Low	Low
Vasani et al. 2017 [10]	Low	Low	Low	Low	Low	Low	Low	Low
Verbraeken et al. 2002 [11]	Low	Low	Low	Low	Low	Low	Low	Low
Al Bashapshe et al. 2010 [12]	Low	Low	Low	Low	Mod	Low	Low	Low
Di et al. 2008 [13]	Low	Low	Low	Low	Low	Low	Low	Low
Massimi et al. 2011 [14]	Low	Low	Low	Low	Low	Low	Low	Low

Results

Table 2 summarizes the findings of all the included papers. For ease of reading, the data for the primary outcome measures are portrayed in the table below.

**Table 2:** All included papers with a summary of the key findings. PMHx: past medical history; Resp Sx: respiratory symptoms; ACM: Arnold-Chiari malformation; MV: mechanical ventilation; Neuro Sx: neurological symptoms; HCP: hydrocephalus; NIV: non-invasive ventilation

Studies	Age	PMHx/underlying disease	Initial presentation	ACM type	Syringomyelia present	Mechanical ventilation	Extubation failures	Required tracheostomy	Surgical intervention	Survival	Long term ventilation
Kijsirichareanchai et al. 2014 [1]	49	N/A	Resp Sx	1	Y	Y	4	Y	None	Y	MV
Khatib et al. 2020 [2]	37	None	Resp Sx	1	Y	Y	N/A	N	Posterior fossa decompression	Y	None
Wani et al. 2011 [5]	31	None	Resp + Neuro Sx	1	N	Y	N/A	Y	None	Y	MV
Gentry et al. 2001 [6]	38	Heavy smoker	Resp + neurological Sx	1	Y	Y	None	N	Emergency posterior fossa decompression	Y	None
Gladding and Whyte 2005 [7]	22	BMI 60	Resp + neurological Sx	1	Y	Y	N/A	N	Emergency posterior decompression	Y	NIV
Fuller and Stanners 2000 [8]	72	Kyphoscoliosis, non-smoker	Resp Sx	N/A	Y	Y	At least 1	Y	None	Y	None
Ding et al. 2020 [9]	37	BMI 24.6	Resp Sx + neurological Sx	1	N	Y	At least 1	Y	Refused	Y	None
Vasani et al. 2017 [10]	35	None	Resp Sx	1.5	Y	Y	None	N	Foramen magnum decompression	Y	None
Verbraecken et al. 2002 [11]	73	BMI 23, known syringomyelia with kyphoscoliosis	Resp Sx	N/A	Y	Y	At least 1	Y	None	Y	NIV
Al Bashapshe et al. 2010 [12]	40	None	Resp Sx + neurological Sx	N/A	Y	Y	At least 1	Y	None	N/A	MV
Di et al. 2008 [13]	50	Known ACM-1 with previous posterior fossa decompression	Neurological Sx	N/A	N	Y	1	N	Partial suboccipital cranioplasty	Y	None
Di et al. 2008 [13]	66	Known ACM-1 with previous posterior fossa decompression	Neurological Sx	1	N	Y	2	Y	Partial suboccipital cranioplasty	Y	MV
Massimi et al. 2011 [14]	38	Noonan syndrome	Resp Sx	1	Y	Y	N/A	Y	Endoscopic third ventriculostomy (for acute HCP)	Y	None

Patient Demographics

Out of the 13 patients in this review, the mean age was 45.2 years with the majority of adult patients younger than 40 years. Males were nearly twice as common as females, and ethnic origin was recorded in only one study, which included a Saudi female (Table 3) [5].

**Table 3:** Population breakdown by age group, mean age, and sex.

Variables	No. of patients (%)
Patient age (years)	
20-40	8 (61.5)
41-60	2 (15.4)
61-80	3 (23.0)
Mean age	45.2
Patient sex	
Male	8 (61.5)
Female	5 (38.5)

Past Medical and Surgical History

Out of our 13 patients, five had no known co-morbidities, five had known CNS disease/abnormality, one was a smoker [6] and one had an obese BMI (Table 4) [7]. The medical history of one patient was not mentioned.

**Table 4:** Population characterized by documented comorbidities.

PMHx	No. of patients (%)
No other comorbidities	5 (38.5)
Known CNS disease/abnormality	
ACM	2 (15.4)
Syringomyelia	1 (7.7)
Noonan	1 (7.7)
Kyphoscoliosis	1 (7.7)
Other comorbidities	
Smoker	1 (7.7)
BMI >30	1 (7.7)

Initial Presenting Symptoms

Patients either presented with respiratory, neurological symptoms, or both. This included patient shortness of breath and headaches in the majority of cases. Three patients had respiratory arrests in the ED requiring immediate intubation and ventilation and over half the patients had mild-to-severe respiratory acidosis as indicated by their blood gas analyses (Table 5) [1,2,8].

**Table 5:** Breakdown of patient symptoms at presentation. Resp: respiratory; neuro: neurological

Symptoms at presentation	No. of patients (%)
Respiratory symptoms	6 (46.2)
Neurological symptoms	2 (15.4)
Resp + neuro symptoms	5 (38.5)
Respiratory acidosis	7 (53.8)
Respiratory arrest	3 (23.1)

Diagnostic Interventions 

Seven patients had a recorded chest plain radiograph (CXR), with four indicating pulmonary infiltrates, and two of these patients had CT chest imaging confirming consolidated lung [9,10]. Two patients needed a bronchoalveolar lavage (BAL) but were negative for any microorganisms. Only one patient had an abnormal transthoracic echocardiogram (TTE) showing a "floppy mitral valve" [11] and one patient had a magnetic resonance angiography (MRA) of the spine showing a spinal artery thrombosis [5]. All patients had a formal diagnosis of neurological pathology based on an MRI of the head and spine, but only four patients had a CT scan of the head mentioned and reported. Two patients had normal CSF from lumbar punctures [5,12] and two patients had an electroencephalogram (EEG) and electromyogram (EMG) which were reported as no abnormality detected (NAD) (Table 6) [1,8].

**Table 6:** All diagnostic interventions recorded. BAL: bronchoalveolar lavage; TTE: transthoracic echocardiogram; CXR: chest plain radiograph; EMG: electromyogram; EEG: electroencephalogram

Diagnostic tests	Total number of patients (%)	No. of patients with normal test	No. of patients with abnormal test
CXR	7 (53.8)	3	4
ECG	5 (38.5)	5	0
TTE	2 (15.4)	1	1
Positive microbiology			
Influenza A	1 (7.7)	0	1
Rhinovirus	1 (7.7)	0	1
Lumbar puncture	2 (15.3)	2	0
CT head	4 (31.0)	0	4
MRI head/spine	13 (100)	0	13
MRI angiogram	1 (7.7)	0	1
Other			
CT chest	3 (23.1)	1	2
BAL	2 (15.4)	2	0
EEG	1 (7.7)	1	0
EMG	1 (7.7)	1	0

Further Diagnostic Interventions: Arnold-Chiari Malformation (ACM) Type and Syringomyelia Presence

Most of the patients had a confirmed type 1 ACM with one patient having a Chiari 1.5 malformation which is a progression of type 1 with caudal herniation of the brainstem through the foramen magnum [10]. Nine patients had congenital syringomyelia confirmed on MRI with six of these associated with ACM as well. Out of these nine syrinxes, five were contained within the cervical spine with two extending inferiorly to include the thoracic spine and two extending superiority to include the cervico-medullary junction (Table 7).

**Table 7:** ACM type and syringomyelia presence + location. ACM: Arnold-Chiari malformation

Variables	No. of patients (%)
ACM	
Type 1	8 (61.5)
Type 1.5	1 (7.7)
N/A	4 (31.0)
Syringomyelia	
Yes	9 (69.2)
No	4 (15.4)
ACM + syringomyelia	6 (46.1)
Congenital syringomyelia location	
Extending to cervicomedullary junction	2 (15.4)
Including cervical spine alone	5 (38.5)
Including cervical + thoracic spine	2 (38.5)

Surgical Intervention

Seven patients underwent a surgical intervention with four patients having an emergency posterior fossa decompression, two having a suboccipital cranioplasty after previous failed decompressions [13], and one patient needing an endoscopic third ventriculostomy (ETV) [14]. One patient refused surgery and no patients had any major intraoperative complications documented [9]. All seven patients had post-operative imaging (CT/MRI), in which six of the patients reported the following expected post-operative changes: adequate decompression, decrease in syringomyelia/ventricular size, and no hydrocephalus, infarction, or bleeding (Table 8).

**Table 8:** Surgical intervention used and post-operative factors.

Surgical procedure	No. of patients (%)
Total	7 (100)
Decompression	4 (57.1)
Cranioplasty (after previous decompression)	2 (28.6)
Endoscopic third ventriculostomy	1 (14.3)
Refused	1
Redo/revision	0
Intraoperative complications	None
Adequate post-operative imaging reported	7 (100)

Critical Care Morbidity and Mortality Factors

Table 9 presents our ventilation data, showing that all patients were mechanically ventilated, with five patients also requiring NIV during their length of stay. Eight patients had tracheostomies, and we had extubation data for nine patients. Of which, two patients had a successful extubation and five patients had one or more extubation failures which were due to apnea, respiratory distress, and poor secretion control. One patient had two extubation failures [13] and another patient had four extubation failures recorded [1].

**Table 9:** Ventilation types and extubation failures.

Variables	No. of patients (%)
Ventilation type	
Invasive mechanical ventilation	13 (100)
Non-invasive ventilation	5 (38.5)
Tracheostomy required	8 (61.5)
Extubation failures	
0	2 (22.2)
1	5 (55.6)
2	1 (11.1)
>2	1 (11.1)

There were no reports of death of the 13 patients in the literature, with nine patients surviving up to 28 days and the rate of survival gradually decreasing with time with one patient surviving up to three years (Table 10) [14]. Four patients had no survival data recorded.

**Table 10:** Survival and mortality data.

Survival	Total number of patients (%)
28-day survival	9 (69.2)
90-day survival	5 (38.5)
6-month survival	4 (30.8)
1-year survival	3 (23.1)
2-year survival	2 (15.4)
3-year survival	1 (7.7)
Dead	0
N/A	4 (30.8)

Data on length of stay (LoS) was variable and predominantly had the minimum length of stays of patients (i.e., what was documented), not the total (Table 11). The majority of patients had a minimum stay of less than 10 days, while two patients had stays of 104 days and six months, respectively, due to prolonged ventilation weans [8,11].

**Table 11:** Length of stay (LoS) of patients.

Minimum length of stay recorded	No. of patients (%)
1-5 days	9 (69.2)
6-10 days	8 (61.5)
11-28 days	4 (30.8)
1-3 months	0
3-6 months	2 (15.4)
>6 months	0
Not recorded	4 (30.8)

Inpatient complications were as follows: seven patients needed emergency reintubation due to respiratory decompensation (as previously described) with three patients being ventilator-dependent for more than 30 days and needing an extensive wean (Table 12). One patient was documented as going into multi-organ failure [5], although descriptions of which organs were supported were not available and another patient required a percutaneous endoscopic gastrostomy (PEG) tube for long-term enteral nutrition [13].

**Table 12:** Inpatient and long-term complications.

Variables	No. of patients (%)
Inpatient complications	
Reintubation	7 (53.8)
Multi-organ failure	1 (7.7)
PEG insertion	1 (7.7)
Extensive wean from ventilator (>30 days)	3 (23.1)
Long-term complications (after 30 days)	
Recurrence of respiratory symptoms	5 (38.5)
Recurrence of neurological symptoms	1 (7.7)
Long-term mechanical ventilation	3 (23.1)
Long-term non-invasive ventilation	2 (15.4)
Readmission	0

Long-term complications included five patients with recurrent respiratory symptoms as follows: two patients with ongoing obstructive sleep apnea (OSA) requiring long-term continuous positive airway pressure (CPAP) and three patients becoming long-term ventilator dependent. One patient (the one who refused neurosurgical intervention) continued to have ongoing neurological symptoms including motor and sensory deficits. No patients were reported to be readmitted.

Discussion

Regarding our outcome measures, patient demographics evidently showed that patients were mainly young males; however, only one paper showed ethnicity data, so further data inference in this area could not be drawn. Patient comorbidities were better documented with three patients having known ACM/syringomyelia but the majority being newly diagnosed. Interestingly one patient had Noonan syndrome which is associated with ACM in some case reports [14]. We broke down and categorized the initial presenting symptoms as respiratory and/or neurological in origin with most of the patients presenting with a joint picture. Three patients had a sudden respiratory arrest in ED requiring immediate intubation and ventilation (I&V) with two of these having normal post-arrest CT head (CTH) and one having diffuse cerebral edema; however, CT was not diagnostic for the cause of the cardiac arrest. Further diagnostic interventions were needed and all patients underwent an MRI head and spine to definitively diagnose ACM and/or syringomyelia. One patient (CTH - diffuse cerebral edema) needed an MRA spine, which described a spinal artery thrombosis that explain their deterioration and subsequent multi-organ failure. Other additional tests, such as lumbar puncture (LP), EEG, and EMG, were done but were not diagnostic.

There are four types of Arnold-Chiari malformations (ACM) described in the current literature with type 1 being the most common with a prevalence of one in 1000 births [1]. Type 1 ACM leads to the cerebellar tonsils herniating inferiorly below the level of foramen magnum, which eight of our 13 patients were diagnosed with [2]. Neurosurgery is indicated when the patient has progressive neurological deficits (sensory and motor), increasing syringomyelia (which has likely caused respiratory decompensation), or unmanageable symptoms. Syringomyelia incidence in patients with ACM is estimated to be around 40% in children and 69% in adults; however, the prevalence in our subset of patients was lower (46%) [15,16]. Syringomyelia can also be caused by spinal trauma, malignancies, and infection, but was out of the remit of this paper. The symptoms associated with syringomyelia are dependent on its location (cervical to sacral) and the position of the cavity (unilateral to diffuse) which can lead to mild symptoms, such as a distorted sensorium or severe complete paralysis associated with spasticity [16]. All nine patients with syringomyelia had their syrinx contained within the cervical spine specifically within C2, which is likely why they had a respiratory deterioration due to compromised diaphragmatic function from neuromuscular failure [1]. Furthermore, Gowers mentions a cape-like loss of sensation (predominantly in the shoulder region) associated with syringomyelia as it grows within the spinal canal, which is similar to the presentation of central cord syndrome [17]. Thus, if suspecting syringomyelia, a thorough neurological examination should be completed; however, they can present with mixed upper motor and lower motor neuron signs, especially if multiple pathologies are present [15]. Furthermore, it can be made more difficult if the patient is sedated and ventilated in intensive care which can mask clinical signs. Therefore, it is best done in patients off sedation, but if this is not possible then consulting specialist neurologist opinions alongside examination can guide imaging modality choice and further management.

Posterior fossa decompression is usually first-line surgery which improves CSF flow by relieving pressure at the craniocervical junction which should subsequently improve respiratory and neurological symptoms. This is paralleled with four out of the seven patients who were operated on having this procedure. Gagnadoux et al. reported a significantly reduced central apnea index (using polysomnography) after decompression surgery in ACM patients [15]. However reconstructive cranioplasty was required in two of our patients after failed decompression due to their ongoing neurological symptoms, which included intractable headaches and gait imbalance. In one patient with a holocord syrinx and chronic supratentorial hydrocephalus, an ETV was required primarily to relieve the hydrocephalus while diverting CSF [14]. All our surgical patients had documented adequate post-operative CTH with no further infarcts, bleeds, or surgical complications, with five patients having a resolution of respiratory/neurological symptoms and no ventilator dependence. Additionally, the literature, also mentions the insertion of different types of syrinx shunts (syringo-subarachnoid, syringo-peritoneal, syringo-pleural) to relieve symptomatic syringomyelia if initial decompression is unsuccessful, with syringo-pleural shunts having the best clinical outcomes and the lowest revision rates [18].

Critical care morbidity and mortality data were inconsistent in our review, and survival and LoS data were difficult to interpret because we only had the minimum time recorded, not the total time. Thus, although no deaths were recorded, less than half the patients had outcome data over one year since their initial diagnosis/presentation. Also, half of the included papers did not have data for the number of ventilator days, so this was not analyzed. However, we were able to interpret other ventilation data, eight patients had tracheostomies due to repeated extubation failures which were primarily due to acute respiratory distress, apnea, and worsening secretion burden. This was most likely caused by ongoing neuromuscular weakness of the high cervical syrinx and pressure at the cervico-medullary junction [1]. Inpatient complications included one patient who was fed via a percutaneous endoscopic gastrostomy (PEG) tube [13], though no further details were provided. Another patient developed multi-organ failure due to a spinal artery thrombosis, which caused severe cord and cerebral edema, ultimately resulting in ventilator dependency and a vegetative state [5].

Our patient subset also had worsened morbidity due to ongoing ventilatory failure and dependency. Five patients in total needed long-term non-invasive or invasive ventilation. This included two of our seven post-operative patients who still required CPAP and mechanical ventilation, respectively, for ongoing respiratory failure, which is further supported by Paul et al., who state that although in post-operative decompressed ACM patients there is initial resolution of symptoms, 21% show a symptom relapse within two to three years post-procedure [19]. This is likely due to syrinx and CSF reaccumulation which leads to further compression within the spinal canal and cranial junction [20].

## Conclusions

ACM and congenital syringomyelia lead to acute respiratory failure and related extubation failures. Our review indicates that these patients are more likely middle-aged males who present with a mix of respiratory and neurological symptoms. Type 1 ACM and cervical syringomyelia are the most common types/locations in this patient subset. Neurosurgical intervention including by majority posterior fossa decompression is indicated to relieve neurological symptoms and aims to liberate patients from the ventilator. However, our scope of the current literature suggests patients still undergo long ventilation weans and require long-term ventilation due to ongoing respiratory failure despite surgery. This worsens patient morbidity significantly due to greater length of stay, high tracheostomy rates, and recurrence of respiratory symptoms, yet, patient mortality is very low. This systematic review revealed gaps in key outcome data, including length of stay and ventilator days. Although this is a rare neurological pathology, we recommend further high-level research to evaluate these patients' presentations and the sequelae of their disease burden.
